# Enhanced Fenton Degradation of Tetracycline over Cerium-Doped MIL88-A/g-C_3_N_4_: Catalytic Performance and Mechanism

**DOI:** 10.3390/nano14151282

**Published:** 2024-07-30

**Authors:** Abdelazeem S. Eltaweil, Amira M. Galal, Eman M. Abd El-Monaem, Nouf Al Harby, Mervette El Batouti

**Affiliations:** 1 Department of Engineering, Faculty of Engineering and Technology, University of Technology and Applied Sciences, Ibra 400, Oman; abdelazeemeltaweil@alexu.edu.eg; 2 Department of Chemistry, Faculty of Science, Alexandria University, Alexandria 21934, Egypt; amira.galal@alexu.edu.eg (A.M.G.); emanabdelmonaem5925@yahoo.com (E.M.A.E.-M.); mervette.elbatouti@alexu.edu.eg (M.E.B.); 3 Department of Chemistry, College of Science, Qassim University, Buraidah 51452, Saudi Arabia

**Keywords:** Ce-doping, GC-MS, degradation mechanism, Fenton-like degradation, XPS

## Abstract

Since enormous amounts of antibiotics are consumed daily by millions of patients all over the world, tons of pharmaceutical residuals reach aquatic bodies. Accordingly, our study adopted the Fenton catalytic degradation approach to conquer such detrimental pollutants. (Ce_0.33_Fe) MIL-88A was fabricated by the hydrothermal method; then, it was supported on the surface of g-C_3_N_4_ sheets using the post-synthetic approach to yield a heterogeneous Fenton-like (Ce_0.33_Fe) MIL-88A/10%g-C_3_N_4_ catalyst for degrading the tetracycline hydrochloride drug. The physicochemical characteristics of the catalyst were analyzed using FT-IR, SEM-EDX, XRD, BET, SEM, and XPS. The pH level, the H_2_O_2_ concentration, the reaction temperature, the catalyst dose, and the initial TC concentration were all examined as influencing factors of TC degradation efficiency. Approximately 92.44% of the TC was degraded within 100 min under optimal conditions: pH = 7, catalyst dosage = 0.01 g, H_2_O_2_ concentration = 100 mg/L, temperature = 25 °C, and TC concentration = 50 mg/L. It is noteworthy that the practical outcomes revealed how the Fenton-like process and adsorption work together. The degradation data were well-inspected by first-order and second-order models to define the reaction rate. The synergistic interaction between the (Ce_0.33_Fe) MIL-88A/10%g-C_3_N_4_ components produces a continuous redox cycle of two active metal species and the electron-rich source of g-C_3_N_4_. The quenching test demonstrates that ^•^OH is the primary active species for degrading TC in the H_2_O_2_–(Ce_0.33_Fe) MIL-88A/10%g-C_3_N_4_ system. The GC-MS spectrum elucidates the yielded intermediates from degrading the TC molecules.

## 1. Introduction

There is no doubt that water is a lifeline for everyone. Water contamination has progressed significantly in the last few decades with restricted water supplies, making researchers concerned about wastewater treatment. Water pollution describes the contamination of water bodies by potentially dangerous substances, such as industrial and agricultural waste effluents that contain heavy metals, aromatic compounds, dyes, pesticides, and pharmaceuticals [1]. Pharmaceutical pollutants have risen, especially during the COVID-19 pandemic, which has revealed unexpected threatening effects due to the rapid rise in the number of COVID-19 patients who need antibiotics. There has been a corresponding increase in the presence of these drugs, which are improperly disposed of through sewage or medical waste [2]. These pharmaceutical contaminants seriously impede human health and aquatic life to a frightening degree [3]. Consequently, researchers have presented efforts to conquer this inevitable danger [4].

Tetracycline hydrochloride (TC) is a broad-spectrum antibiotic utilized for treating bacterial infections, including acne, urinary tract infections, respiratory tract infections, and skin, stomach, and intestine infections. Notably, TC is present in high concentrations because it is not entirely digestible, and between 50 and 80 percent of the dosage is excreted in the urine [2]. Thus, TC residues end up in water bodies, increasing the chances of bacteria developing antibiotic resistance [5]. Thereby, to prevent this, a variety of remediation methods have been developed for the elimination of TC from wastewater, including membrane separation [6], microbial degradation [7], electro-oxidative degradation [8], photocatalysis [9], adsorption [10], and advanced oxidation processes (AOPs) [11]. Among these methods, AOPs have drawn particular attention from researchers due to their eco-friendliness and high efficiency and reproductivity in eliminating TC [12,13].

In 1894, Fenton disclosed the Fenton reaction, which aims to convert pollutants to less harmful substances [13]. The conventional homogeneous Fenton reaction system is a mixture of Fe^2+^ and H_2_O_2_ in highly acidic conditions to generate Fe^3+^ and hydroxyl radicals ^⦁^OH with a high redox potential (E^o^ of ^⦁^OH/H_2_O_2_ = 2.73 V) as the main reactive oxygen species (ROS) to degrade contaminants in a non-selective way [11,14]. Then, the reaction between Fe^3+^ and H_2_O_2_ reduces it to Fe^2+^, so ^⦁^OH will be produced endlessly by a continuous redox cycle (Fe^2+^/Fe^3+^) [13,15]. The traditional Fenton reaction has flaws such as not being recyclable, a large consumption of H_2_O_2_, acidic pH, and iron leaching. Consequently, the insoluble solid catalyst-based heterogeneous Fenton-like reaction has been developed to enhance the issues mentioned above since it is inexpensive, has mild operating conditions, recovers quickly, and does not produce sludge [12,14,16,17]. The Fenton-like catalyst is an insoluble solid catalyst that imitates the behavior of the Fenton reaction. Furthermore, the Fenton-like catalyst can include other non-ferrous multivalent transition metals (Ni, Co, Ag, and Au), metal oxides (Mn_3_O_4_, NiS, and CeO_2_), carbon-based materials (activated carbons, carbon nanotubes, graphite, graphene and reduced graphene oxide) as well as other new type materials (e.g., polyoxometalates and metal–organic frameworks, MXene, and layered double hydroxide) [18,19]. On the other hand, adsorption is a potent approach because of its distinctive benefits, including being simple, economical, energy-saving, and environmentally friendly, and its ease of regeneration and high efficiency [20]. According to pioneering studies, the Fenton process and adsorption work together to improve removal efficiency for organic contaminants [21].

Porous materials comprising metal–organic frameworks (MOFs) have exhibited great interest as super-eminent adsorbents for purifying organic contaminant-containing wastewater. Metal–organic frameworks are engineered from metal ions bound by organic ligands. Noteworthy, MOFs are advanced substances with promising features, such as water stability, high thermal and chemical stability, and functionalization simplicity. Also, MOFs have a large surface area, a variable pore size, and elastic interior surface features. These properties make them valuable in numerous applications, including gas storage, separation, sensors, solar cells, batteries, supercapacitors, drug delivery, membranes, adsorption, and catalysis [22,23]. Until now, the Fe-based MIL family, which comprises MIL-88A(Fe), MIL-88B(Fe), MIL-53(Fe), MIL-100(Fe), and MIL-101(Fe), has demonstrated significant promise as a successful Fenton-like catalyst [24,25]. Strikingly, MIL-88A(Fe) is the most widely used since it is easy to produce and has strong chemical and water stability [12]. Also, it is environmentally friendly because it can be prepared in an aqueous medium instead of a toxic solvent like dimethyl formamide [26]. Since MIL-88A(Fe) only includes Fe^3+^ species with a weak Fenton degradation aptitude, it is necessary to enhance the catalytic effectiveness of the pure MIL-88A(Fe) [13]. Adjusting the Lewis acidity of MIL-88A(Fe) through cerium (Ce) doping may result in more intrinsic ligand missing defects and improve the catalytic activity. Cerium is one of the most common rare-earth elements that is characterized by its rich redox characteristics, high electronic/ionic conductivity, adaptable coordination ability, and hard Lewis acid [27,28,29].

Carbon nitride is a 2D polymer with a layer-like structure that can be synthesized easily from cheap and abundant resources. g-C_3_N_4_ is a stable, low-cost, non-toxic, high thermal and chemical stability, and environmentally friendly material. Studies implied the favorability of using g-C_3_N_4_ as a Fenton-like catalyst during the degradation of organic pollutants. The g-C_3_N_4_ catalyst can activate hydrogen peroxide to generate hydroxyl radicals, which can then oxidize the organic contaminants. The activity, stability, accessibility of active sites, and surface area of g-C_3_N_4_ as a Fenton-like catalyst can be boosted by doping it with other elements such as iron or cobalt or by coupling with different materials like metal oxides, graphene, and MOF [30,31,32,33].

Herein, we report the synthesis of a novel heterogeneous Fenton-like catalyst for degrading the residual TC molecules in wastewater. The Fenton-like catalyst was fabricated by doping the Ce species onto the MIL-88A surface, and then the obtained Ce-doped MIL-88A was combined with g-C_3_N_4_. To optimize the best molar ratios between the catalyst components, a series of synthesis experiments were performed to fabricate Ce-doped MIL-88A/g-C_3_N_4_ with various molar ratios of the doped Ce species and g-C_3_N_4_. The as-fabricated (Ce_0.33_Fe) MIL-88A/10%g-C_3_N_4_ catalyst was fully characterized via XRD, XPS, SEM, SEM-EDX, BET, elemental mapping and FT-IR to investigate its structural, compositional, and morphological specifications. After several lab experiments, the best conditions for the Fenton-like reaction of TC by the H_2_O_2_–(Ce_0.33_Fe) MIL-88A/10%g-C_3_N_4_ system were thoroughly determined. The potential degradation pathway of TC was supposed by the experimental results of the scavenging test and the XPS analysis. Furthermore, the stability of the (Ce_0.33_Fe) MIL-88A/10%g-C_3_N_4_ catalyst was assessed by performing a recycling test and defining the metal leaching concentration. The intermediate molecules during the Fenton-like degradation of TC by (Ce_0.33_Fe) MIL-88A/10%g-C_3_N_4_ were determined utilizing GC-MS.

## 2. Experimental

### 2.1. Materials

Ferric chloride hexahydrate 97% (FeCl_3_·6H_2_O) and Fumaric acid (C_4_H_4_O_4_) 99.5% were acquired from Loba Chemie (Mumbai, India). Cerium oxalate, sodium hydroxide (97%, NaOH), hydrochloric acid (37%, HCl), and ethanol (99%, C_2_H_5_OH) were purchased from Merck, Rahway, NJ, USA. P-benzoquinone (PBQ) (99%) was supplied from Shanghai Chemical Reagent Co., Ltd. (Shanghai, China). Urea CO(NH_2_)_2_, Hydrogen peroxide (50%), and tetracycline hydrochloride (C_22_H_25_Cl N_2_O_8_, ≥ 99%, MW 480.9 g/mol) were provided by Sigma-Aldrich Co. (Darmstadt, Germany). Without any additional purification, all chemicals employed in the synthesis steps were used as obtained.

### 2.2. Synthesis of MIL-88A

The hydrothermal technique was utilized to manufacture MIL-88A [34]. Briefly, 10 mmol of FeCl_3_.6H_2_O and 10 mmol of Fumaric acid were dissolved into 50 mL ultrapure water and vigorously stirred using a magnetic stirrer for nearly 1 h at room temperature. The mixture was then poured into a 100 mL autoclave and heated in an oven for 24 h at 85 °C. After cooling down to ambient temperature without any external assistance, the obtained orange precipitate was collected using the centrifuge, purified using ethanol and deionized water for one time, and dried at 85 °C overnight.

### 2.3. Preparation of Ce-Doped MIL-88A

The same former procedure was applied to fabricate the Ce-doped MIL-88A(Fe) materials with an additional step, which included adding various amounts of cerium oxalate ranging from 5 to 50%. The Ce species was mixed to FeCl_3_.6H_2_O under stirring for 1 h, and then the rest of the preparation procedure of MIL-88A was completed as previously mentioned.

### 2.4. Synthesis of g-C_3_N_3_

To prepare the sheet-like g-C_3_N_4_, 10 g of urea was placed in a 200 mL closed porcelain crucible. The crucible was then calcinated in a muffle furnace for 4 h at a temperature of 550 °C with a heating rate of 2 °C/min. The black powder of g-C_3_N_4_ was stored in a glass tube until used in the catalyst fabrication.

### 2.5. Synthesis of Ce-Doped MIL-88A/g-C_3_N_4_ Composite

**Scheme 1:**
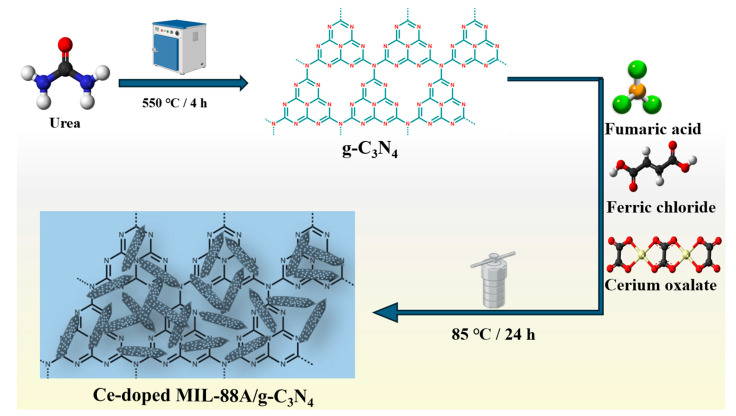
The simple representation of the synthesis process of (Ce_0.33_Fe) MIL-88A/10%g-C_3_N_4_.

The fabrication process of (Ce_0.33_Fe) MIL-88A/10%g-C_3_N_4_ is clarified in Scheme 1. The Ce-doped MIL-88A/g-C_3_N_4_ composite was prepared as follows: 7.5 mmol of FeCl_3_.6H_2_O, 2.5 mmol of cerium oxalate, and 10.0 mmol of C_4_H_4_O_4_ were added separately to 50 mL of ultrapure water and stirred for almost an hour at room temperature. Next, a specific mass ratio of g-C_3_N_4_ ranging from 5 to 20 wt.% was sonicated for 30 min in 10 mL of ultrapure water. The g-C_3_N_4_ suspension was gradually added over the Ce/Fe/C_4_H_4_O_4_ solution. The resultant mixture solution was agitated for an additional hour, put into a 100 mL autoclave, and heated for 24 h at 85 °C in the oven. The obtained precipitate was separated by centrifuge after naturally cooling to ambient temperature, purified once with ethanol and distilled water, and then left to dry at 85 °C overnight.

### 2.6. Characterization

The catalysts’ crystal structure and surface functional groups were analyzed using powder X-ray diffraction (XRD-Bruker D8 Advance, Burker, MA, USA), and Fourier-transform infrared spectra (FTIR-Bruker Equinox 55). X-ray photoelectron spectroscopy (XPS-Thermoscientific-Escalab-250Xi VG) investigated the elemental states of samples. The samples’ morphology and microstructure were analyzed using scanning electron microscopy and energy dispersive X-ray microscopy (SEM, SEM-EDX-JSM-760F). The gas chromatography–mass spectrometry (SHIMADZU, GCMS-QP 2010 Ultra) technique investigated the degradation routes.

### 2.7. Fenton-like Degradation Experiments

TC was degraded in a 50 mL covered container with aluminum foil to avoid the influence of the light on degrading the TC molecules. An amount of 0.01 g of (Ce_0.33_Fe) MIL-88A/10%g-C_3_N_4_ was soaked into the TC solution (50 mg/L, 20 mL) with continuous stirring. After attaining the state of adsorption–desorption equilibrium, H_2_O_2_ was added to the TC-(Ce_0.33_Fe) MIL-88A/10%g-C_3_N_4_ adsorption system. The effects of experimental factors were examined at wide ranges, including the initial concentration of TC (50–300 mg/L), H_2_O_2_ concentration (10–200 mg/L), temperature (25–55 °C), catalyst dosage (0.005–0.02 g), and pH medium (3–11). During the degradation, a sample was collected and analyzed at intervals times. The TC concentration was investigated via an ultraviolet–visible spectrophotometer at 354 nm. Each experiment was conducted in triplicate to ensure the accuracy and reproducibility of the results. The average value and standard deviation (represented by error bars) were calculated for each set of data. The TC removal rate was calculated as follows:

(1) $$Removal\;efficiency\;\%=\frac{C_{0}-C_{t}}{C_{0}}\times 100\%$$

C_0_ = the TC initial concentration, and C_t_ = the TC concentration at a specific time during the Fenton degradation.

### 2.8. Quenching Test

Scavengers like tert-butanol (TBA) and chloroform (CF) were added separately to the catalytic system to determine the radical species and define the controlling radicals in the reaction. After adjusting the pH of the solution, 0.01 g of the catalyst, 10 mL of scavenger, and H_2_O_2_ were added sequentially to 20 mL of TC (50 mg/L). Finally, a sample was taken during the degradation process and examined periodically.

### 2.9. Regeneration Test

The regeneration test was conducted on the (Ce0_.33_Fe) MIL-88A/10%g-C_3_N_4_ catalyst for five Fenton-like degradation cycles of the TC molecules. After each catalytic cycle, (Ce_0.33_Fe) MIL-88A/10%g-C_3_N_4_ was collected by centrifugation and soaked in 20 mL of NaOH solution with a concentration of 0.01 M for 100 min. Next, the catalyst was separated, washed with distilled H_2_O, and dried at 50 °C for reuse in the sequential catalytic cycle.

## 3. Results and Discussion

### 3.1. Characterization of (Ce_0.33_Fe) MIL-88A/10%g-C_3_N_4_

#### 3.1.1. Fourier-Transform Infrared Spectra (FT-IR)

**Figure 1:**
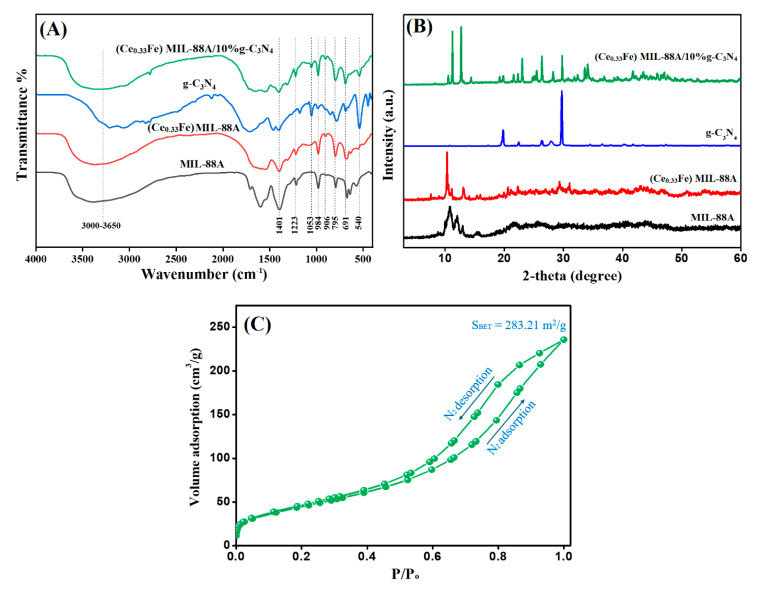
(**A**) FTIR, (**B**) XRD of MIL-88A, (Ce_0.33_Fe) MIL-88A, g-C_3_N_4_, and (Ce_0.33_Fe) MIL-88A/10%g-C_3_N_4_, and (**C**) N_2_ adsorption/desorption hysteresis loop of (Ce_0.33_Fe) MIL-88A/10%g-C_3_N_4_.

FT-IR spectra were applied to explicate the chemical bonding and molecular structure of MIL-88A, (Ce_0.33_Fe) MIL-88A, g-C_3_N_4_, and (Ce_0.33_Fe) MIL-88A/10%g-C_3_N_4_, as illustrated in Figure 1A. The broader absorption band of MIL-88A around 3000–3650 cm^−1^ was assigned to the O-H stretching vibrations of water adsorbed on the catalyst’s surface. The peaks at 1600.22 and 1396.47 cm^−1^, respectively, were caused by the asymmetric and symmetric vibrations of the carboxyl group (-COOH) of the fumaric acid [26]. The stretching vibrations of C=O, C-C, Fe-O, and the bending vibration of C-H were responsible for characteristic peaks at 1708.57, 1216.64, 572.23, and 980.29 cm^−1^, respectively [35]. In the (Ce_0.33_Fe) MIL-88A, the redshift of the Fe-O peak to a shorter wavelength (556.20 cm^−1^) may be explained by the Ce doping and the Ce–O–Fe formation [29]. The relatively large peaks in g-C_3_N_4_ between 3210.77 and 3062.21 cm^−1^ are assigned to the bending vibration of terminal NH_2_ or NH groups [36]. The belonging peaks of the stretching vibrations of the heterocyclic C-N and C=N groups appeared between the wavenumbers of 1720.02 and 1179.06 cm^−1^. In addition, the peak at 782.62 cm^−1^ resulted from the heptazine ring bending vibration [37]. After the combination of (Ce_0.33_Fe) MIL-88A with g-C_3_N_4_, the spectral lines of these composites all match the absorption band of (Ce_0.33_Fe) MIL-88A and g-C_3_N_4_.

#### 3.1.2. X-ray Diffraction (XRD)

The crystallographic characters of MIL-88A, (Ce_0.33_Fe) MIL-88A, g-C_3_N_4_, and (Ce_0.33_Fe) MIL-88A/10%g-C_3_N_4_ were identified by XRD analysis, determining their average crystallite sizes using the Scherrer equation (Figure 1B). For MIL-88A, the diffraction peaks at 2θ = 10.79°, 11.99°, 15.39°, 21.44°, and 12.93°, respectively ascribed by (100), (101), (012), (022), and (110) planes [13,15,38]. Additionally, the average crystallite size of MIL-88A was 5.93 nm, implying its low crystal size and high surface area. On the XRD pattern of (Ce_0.33_Fe) MIL-88A, it was noticed that the peaks relating to the (011), (100), and (101) crystal planes appeared at the angles of 10.51°, 11.21°, and 12.70°. In addition, there was a noticeable increase in the crystallite size of (Ce_0.33_Fe) MIL-88A (25.43 nm) compared to the pristine MIL-88A, indicating the occurrence of Ce doping. The g-C_3_N_4_ characteristic peaks were found at 2θ = 19.78° and 29.72°, which corresponded to the diffraction planes of (100) and (002), respectively, with an average crystallite size of 42.55 nm [30]. The diffraction peaks for both (Ce_0.33_Fe) MIL-88A and g-C_3_N_4_ were observed in the crystallographic pattern of the (Ce_0.33_Fe) MIL-88A/10%g-C_3_N_4_ composite, indicating that the crystal structures were well retained. The average crystallite size (Ce_0.33_Fe) MIL-88A/10%g-C_3_N_4_ was about 64.27 nm, clarifying an increase in the (Ce_0.33_Fe) MIL-88A size after decorating with g-C_3_N_4_ owes to its large crystallite size.

#### 3.1.3. Brunauer–Emmett–Teller (BET)

The nitrogen adsorption/desorption hysteresis loop of (Ce_0.33_Fe) MIL-88A/10%g-C_3_N_4_ (Figure 1C) clarified the type (VI) isotherm, disclosing the mesoporous structure of the composite. In addition, the derived specific surface area of the (Ce_0.33_Fe) MIL-88A/10%g-C_3_N_4_ catalyst is 283.21 m^2^/g. This result reflects the largely available surface area of the (Ce_0.33_Fe) MIL-88A/10%g-C_3_N_4_ catalyst to proceed the Fenton-like degradation process of TC onto its surface.

#### 3.1.4. X-ray Photoelectron Spectroscopy (XPS)

**Figure 2:**
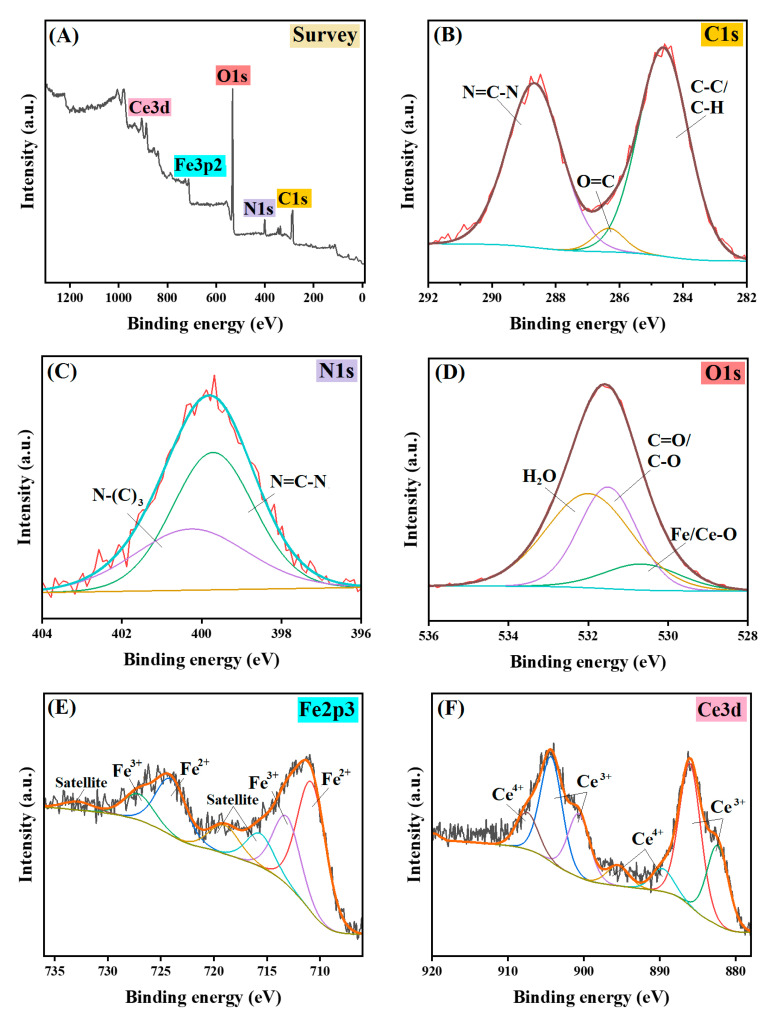
XPS spectra of the (Ce_0.33_Fe) MIL-88A/10%g-C_3_N_4_ catalyst: (**A**) survey, (**B**) carbon, (**C**) nitrogen, (**D**) oxygen, (**E**) iron, and (**F**) cerium.

The (Ce_0.33_Fe) MIL-88A/10%g-C_3_N_4_ catalyst was analyzed using XPS to identify its chemical components and elemental states, as represented in Figure 2. The wide-spectrum (Figure 2A) revealed that (Ce_0.33_Fe) MIL-88A/10%g-C_3_N_4_ is composed of five elements: C, N, O, Fe, and Ce. The C1s spectrum (Figure 2B) exhibited three peaks at 284.62, 288.67, and 286.33 eV, corresponding to sp^2^ hybridized carbon atoms: C-C/C-H bonding, N=C-N bonding, and C=O groups, respectively [18,39]. The N 1s spectrum (Figure 2C) illustrated the related peaks of the tertiary nitrogen (N–(C)_3_) at 400.26 eV and the sp^2^ bonded nitrogen in N-containing aromatic rings (C–N=C) at 399.71 eV [36]. The O 1s peaks (Figure 2D) at 530.65, 531.5, and 531.99 eV are attributed to Fe/Ce-O, C=O/C-O, and water molecule adsorption [40]. The Fe 2p spectrum (Figure 2E) showed the presence of Fe^2+^ 2p3/2 and Fe^2+^ 2p1/2 since the peaks were at 710.79 and 724.04 eV, respectively. Furthermore, the 713.16 and 727.08 eV peaks belong to Fe^3+^ 2p3/2 and Fe^3+^ 2p1/2, respectively. The XPS peaks at 715.65, 719.02, and 732.8 eV are assigned to satellite peaks of 2p3/2 and 2p1/2 [36,41,42]. The Ce 3d peaks (Figure 2F) at 882.38 and 886.02 eV corresponded to Ce^3+^ 3d5/2, while the binding energy at 889.64 and 895.53 eV elucidated Ce^4+^ 3d5/2. Moreover, the Ce^3+^ 3d3/2 appeared at 900.67 and 904.3 eV, and the Ce^4+^ 3d3/2 manifested at 907.47 eV [43,44].

#### 3.1.5. Scanning Electron Microscopy (SEM)

**Figure 3:**
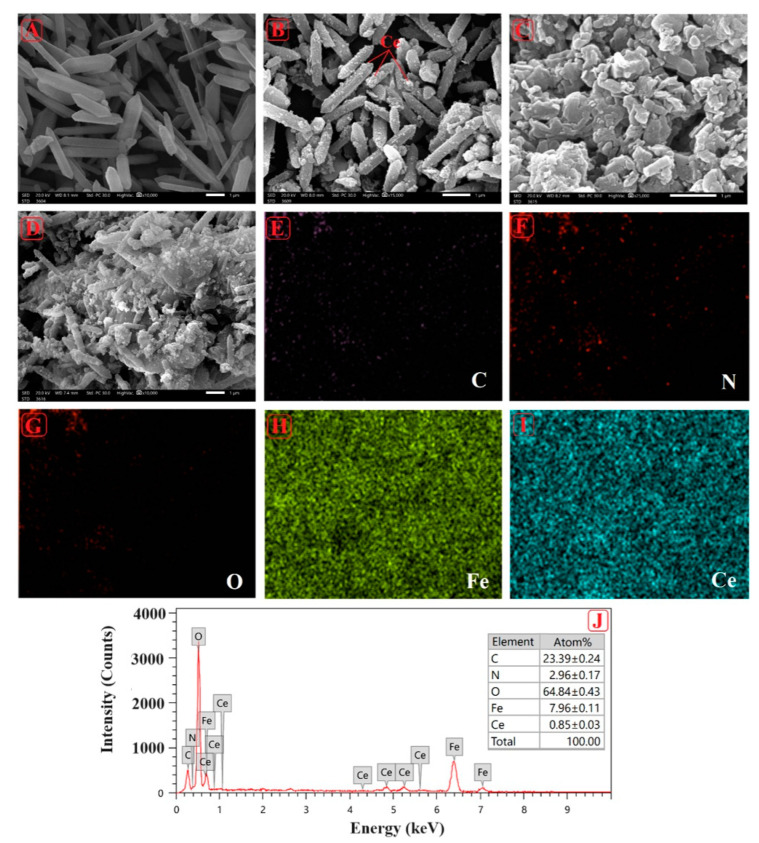
SEM images of (**A**) MIL-88A, (**B**) (Ce_0.33_Fe) MIL-88A, (**C**) g-C_3_N_4_, and (**D**) (Ce_0.33_Fe) MIL-88A/10%g-C_3_N_4_, (**E**–**I**) elemental mapping of (Ce_0.33_Fe) MIL-88A/10%g-C_3_N_4_, and (**J**) SEM-EDX pattern of (Ce_0.33_Fe) MIL-88A/10%g-C_3_N_4_.

In the SEM image (Figure 3A), MIL-88A elucidates a hexagonal structure that looks like a spindle with a very smooth surface, demonstrating the development of a fine crystal structure. The (Ce_0.33_Fe) MIL-88A catalyst (Figure 3B) displays a similar hexagonal structure to MIL-88A with some differentiation in the roughness of the surface and tiny spread particles on the MIL-88A surface. This change in the surface of MIL-88A indicates doping by cerium particles on the MIL-88A surface. The g-C_3_N_4_ image shows smooth and aggregate stacking layers that resemble cracked sheets (Figure 3C). Ultimately, the SEM image of (Ce_0.33_Fe) MIL-88A/10%g-C_3_N_4_ (Figure 3D) demonstrates that (Ce_0.33_Fe) MIL-88A is adhered to the surface of the cracked sheet of g-C_3_N_4_ and continues to have a hexagonal spindle morphology. The elemental mapping images showed the well-distributed Fe, Ce, C, N, and O elements in the (Ce_0.33_Fe) MIL-88A/10%g-C_3_N_4_ composite, as demonstrated in Figure 3E–I. The appearance of the Ce signal indicated the successful doping of cerium species. Additionally, the SEM-EDX pattern denoted that the (Ce_0.33_Fe) MIL-88A/10%g-C_3_N_4_ is composed of carbon, nitrogen, oxygen, iron, and cerium with atomic percentages of 23.39%, 2.96%, 64.84%, 7.96%, and 0.85%, sequentially, as depicted in (Figure 3J).

### 3.2. Fenton-like Degradation of Tetracycline Hydrochloride

#### 3.2.1. Optimization of the Components’ Ratio of Ce-Doped MIL-88A/g-C_3_N_4_

**Figure 4:**
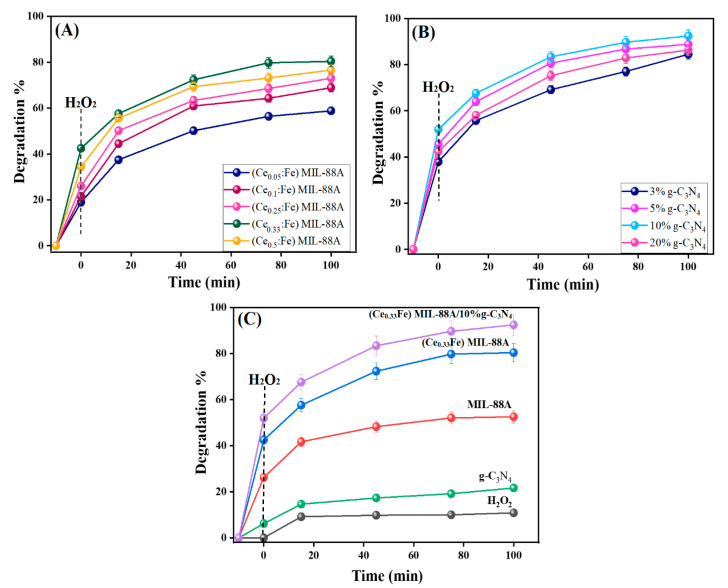
The optimization of (**A**) the Ce-doping proportion and (**B**) the g-C_3_N_4_ proportion in (Ce_0.33_Fe) MIL-88A/g-C_3_N_4_, and (**C**) comparison test between the degradation aptitude of H_2_O_2_, MIL-88A, (Ce_0.33_Fe) MIL-88A, g-C_3_N_4_, and (Ce_0.33_Fe) MIL-88A/10%g-C_3_N_4_ towards the TC molecules.

A series of lab experiments proceeded on TC degradation by Ce-doped MIL-88A/g-C_3_N_4_ to denote the synergistic effect between Ce species, MIL-88A, and g-C_3_N_4_ and identify the optimal Ce-doping proportion and the finest g-C_3_N_4_ amount in the catalyst (Figure 4A–C). The impact of cerium doping on the aptitude of the Fenton-like degradation of TC was investigated by varying the Ce ratio in (Ce_x_Fe) MIL-88A catalysts in which the TC removal efficiency was 58.82%, 68.89%, 73.01%, 80.40%, and 76.58% when the x ratios were 0.05, 0.1, 0.25, 0.33, and 0.5, respectively. This finding suggested that the finest Ce-doping amount in the Ce-doped MIL-88A is x equaled 0.33. This finding could be explained by the essential role the cerium species in activating H_2_O_2_ and yielding ROS radicals, where the Ce^3+^ ions could work with the Fe^2+^ions-containing MIL-88A to expand the redox cycle. Furthermore, the optimal proportion of g-C_3_N_4_ in the (Ce_0.33_Fe) MIL-88A/g-C_3_N_4_ catalysts was determined. The Fenton-like degradation % of TC was 84.49%, 88.78%, 92.44%, and 86.31%, when the g-C_3_N_4_ proportion was 3%, 5%, 10%, and 20%, respectively. This result clarified that the finest g-C_3_N_4_ proportion in the (Ce_0.33_Fe) MIL-88A/g-C_3_N_4_ was 10%. This enhancement in the TC degradation efficiency of Ce_0.33_Fe) MIL-88A after decorating with g-C_3_N_4_ owes to the nitrogen’s lone pairs of electrons that can be used as electron donors since g-C_3_N_4_ itself contains the electron-rich structure of the heptazine ring of the pyridine nitrogen group. So, g-C_3_N_4_ can easily donate electrons to the to activate H_2_O_2_ and recover the catalyst’s metal species. Moreover, it was recorded that the Fenton-like degradation efficacy of MIL-88A, (Ce_0.33_Fe) MIL-88A, g-C_3_N_4_, and (Ce_0.33_Fe) MIL-88A/10%g-C_3_N_4_ towards the TC molecules was 52.51%, 80.40%, 21.64%, and 92.44%, respectively. This observation implied the synergetic effect between the Ce species, MIL-88A, and g-C_3_N_4_ to enhance the produced ROS [18], while in the case of the presence of H_2_O_2_ alone, the TC elimination rate was only 10.83% after 100 min, indicating that H_2_O_2_ is inactive as a catalyst for TC degradation.

#### 3.2.2. Effects of pH

**Figure 5:**
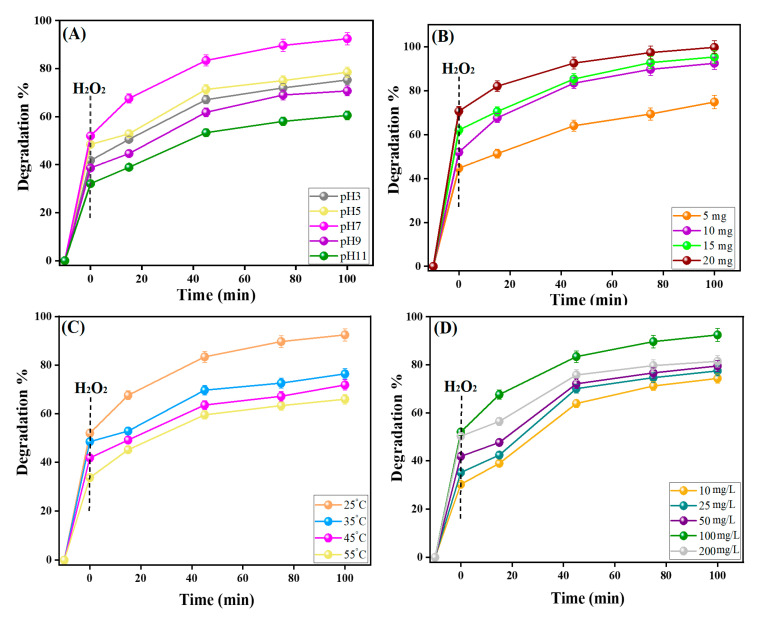
Effects of (**A**) pH, (**B**) catalyst doses, (**C**) temperature, and (**D**) oxidant concentration on the Fenton-like degradation % of TC by (Ce_0.33_Fe) MIL-88A/10%g-C_3_N_4_.

The Fenton-like degradation efficiency of TC was studied on a wide scale of the pH of the catalytic medium, as represented in Figure 5A. As the initial pH was elevated from 3 to 7, the degradation % of TC enhanced drastically to 92.44%. Next, raising the pH medium over pH = 7, the degradation efficiency dropped to 60.51% at pH = 11. These results could be attributed to the lower redox potential of H_2_O_2_ in alkaline media (E^o^ = 1.8 eV) than in acidic media (E^o^ = 2.7 eV). Therefore, at a low catalytic pH media, the amount of the produced ^⦁^OH is low; furthermore, the high concentrations of H^+^ obstruct the ^⦁^OH radicals. Contrariwise, in a low-alkaline and natural media, the higher generated concentrations of ^⦁^OH significantly attacked the TC molecules. In addition, the formed hydro per-oxy anions (OOH^−^) in a high-alkaline medium possess higher affinity than H_2_O_2_ towards the metal species (viz., Ce and Fe), as represented in (Equations (2) and (3)) [45]. Also, auto-decomposition (Equation (4)) is another problem of performing the Fenton-like reaction of TC in a high-alkaline medium [39,46].

H_2_O_2_ + OH^−^→OOH^−^ + H_2_O (2)

M + OOH^−^→M…^•^OOH (3)

M + OOH^−^→O_2_ + 2H_2_O (4)

#### 3.2.3. Effects of Catalyst Dosage

The oxidative degradation of TC by the (Ce_0.33_Fe) MIL-88A/10%g-C_3_N_4_ catalyst can be boosted by increasing the catalyst dosage, so the Fenton-like degradation of TC was studied at varied doses (Figure 5B). As the (Ce_0.33_Fe) MIL-88A/10%g-C_3_N_4_ dosage elevated from 5 to 20 mg/L, the TC elimination rate improved from 74.82% to 99.71% within 100 min. This enhancement can be demonstrated by augmenting the amount of catalyst that promotes the ^⦁^OH radicals and increments specific surface area [16].

#### 3.2.4. Effects of Reaction Temperature

The Fenton-like degradation of TC by the (Ce_0.33_Fe) MIL-88A/10%g-C_3_N_4_ catalyst was examined at various reaction temperatures (Figure 5C). Notably, the TC degradation % diminished from 92.44% to 65.94% as the system temperature elevated from 25 to 55 °C. At low temperatures, TC can be oxidized by ^⦁^OH; but, when temperature increased, ^⦁^OH became more active and began to self-consume, as elucidated in (Equation (5)). Furthermore, H_2_O_2_ was unstable at high temperatures and split into water and oxygen [47].

(5) $${HO}^{•}+{HO}^{•}\to H_{2}O_{2}$$

#### 3.2.5. Effects of H_2_O_2_ Concentration

The TC degradation efficacy by (Ce_0.33_Fe) MIL-88A/10%g-C_3_N_4_ ameliorated obviously from 74.29% to 92.44% with a rise in the H_2_O_2_ concentration from 10 to 100 mg/L because of the ample production of ^•^OH from the high concentration of H_2_O_2_ (Figure 5D) [48]. However, the TC removal slightly dropped to 81.45% by elevating the oxidant concentration to 200 mg/L. This catalytic performance may be due to the high concentration of H_2_O_2_, more than necessary, resulting in scavenged ^•^OH and generating the less active HO_2_^•^ radical, as clarified in Equations (6)–(8) [11].

(6) $$H_{2}O_{2}+{HO}^{•}\to {HO}_{2}^{•}+H_{2}O$$

(7) $${HO}_{2}^{•}+{HO}^{•}\to H_{2}O+O_{2}$$

(8) $${HO}^{•}+{HO}^{•}\to H_{2}O_{2}$$

#### 3.2.6. Effects of Initial Concentration

**Figure 6:**
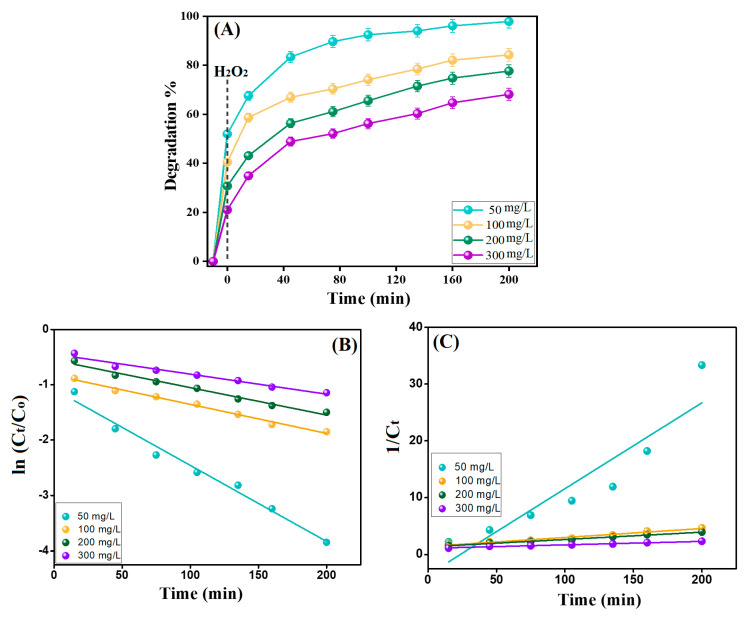
(**A**) The Fenton-like degradation of the TC molecules during 200 min and (**B**) first order and (**C**) second order of TC degradation by H_2_O_2_–(Ce_0.33_Fe) MIL-88A/10%g-C_3_N_4_.

The degradation capability of (Ce_0.33_Fe) MIL-88A/10%g-C_3_N_4_ towards various TC concentrations was examined for 200 min, as depicted in (Figure 6A). Obviously, the adsorption process and Fenton-like reaction showed a synergistic effect, where they worked together to efficiently degrade the noxious TC molecules. During the first step (adsorption), the adsorption percent of TC attained 51.96%, 40.45%, 30.76%, and 21.01% after 10 min, when the TC concentrations were 50, 100, 200, and 300 mg/L. Next, by adding H_2_O_2_ to start the second step (Fenton-like reaction), the TC degradation aptitude reached 97.86%, 84.27%, 77.65%, and 68.15% after 200 min. The results clarified a decline in the degradation efficacy by elevating the TC concentrations, which is most likely due to blocking the active sites of the (Ce_0.33_Fe) MIL-88A/10%g-C_3_N_4_ catalyst, giving rise to the prevention of the generated ROS from being sufficient to remove the excess TC [39].

### 3.3. Kinetic Study

The TC degradation results in the H_2_O_2_–(Ce_0.33_Fe) MIL-88A/10%g-C_3_N_4_ system during 200 min was examined using first-order and second-order models (Equations (9) and (10)).

(9) $$\ln\;\frac{C_{t}}{C_{o}}=-k_{1}t$$

(10) $$\frac{1}{C_{t}}=\frac{1}{C_{o}}+k_{2}t$$

where t = the reaction time, min, k = the rate constant, min^−1^, and C_t_ and C_0_ = the final and initial concentrations of TC, mg/L [39].

In light of the derived R^2^ values from the first-order and second-order plots (Figure 6B,C), the Fenton-like degradation of the TC molecules by (Ce_0.33_Fe) MIL-88A/10%g-C_3_N_4_ was best modeled with the first-order model since its R^2^ values of the whole TC concentrations were higher than the R^2^ of the second-order model (Table 1). Furthermore, the k_2_ of the TC concentrations of 50, 100, 200, and 300 mg/L were 0.1511, 0.0157, 0.0128, and 0.0061 min^−1^.

### 3.4. Identification of Reactive Species

**Figure 7:**
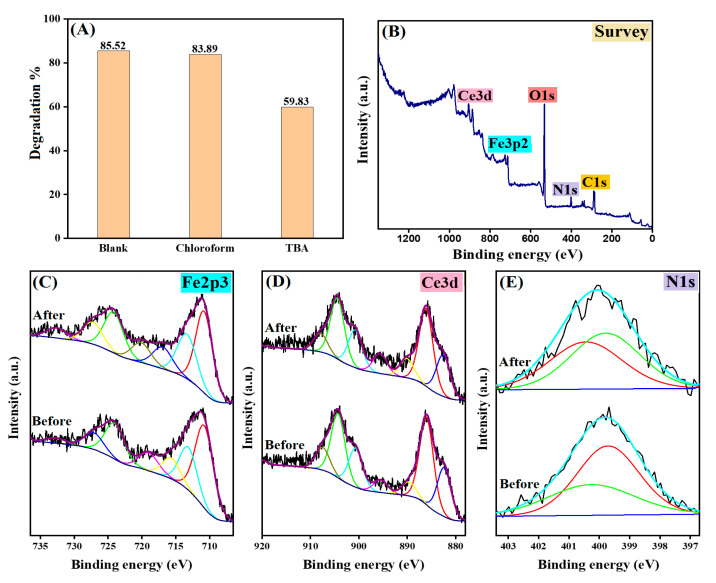
(**A**) Quenching test of the (Ce_0.33_Fe) MIL-88A/10%g-C_3_N_4_ catalyst during the Fenton-like degradation process of TC and XPS of the used (Ce_0.33_Fe) MIL-88A/10%g-C_3_N_4_ catalyst; (**B**) survey, (**C**) Fe2p3, (**D**) Ce3d, and (**E**) N1s.

For identifying the reactive radical species within the TC Fenton-like reaction, the quenching experiment proceeded, individually utilizing tert-butanol (TBA) and chloroform (CF) as scavengers for ^•^OH and ^•^O_2_^−^ species (Figure 7A) [49]. The findings of the scavenging experiments showed that adding TBA to the catalytic medium inhibited the degradation percent of TC, denoting the critical function of ^•^OH in this process. While adding CF does not affect the TC degradation rate, these results indicate that ^•^OH is the leading ROS for TC degradation in the H_2_O_2_–(Ce_0.33_Fe) MIL-88A/10%g-C_3_N_4_ system.

### 3.5. Degradation Mechanism

In light of the experimental findings, TC degraded to less noxious molecules throughout two sequential stages: adsorption and the Fenton-like reaction. The XPS survey of the used (Ce_0.33_Fe) MIL-88A/10%g-C_3_N_4_ catalyst (Figure 7B) demonstrated an increase in the atomic percentages of nitrogen, carbon, and oxygen, confirmed proceeding the adsorption process of TC (first stage). The adsorption of the TC molecules can occur through the formation of H-bonds between oxygen functional groups between (Ce_0.33_Fe) MIL-88A/10%g-C_3_N_4_ and the hydrogen atoms of TC. However, these H-bonds are weaker than those between water molecules and TC. On the other hand, the coordination bonds consist of the oxygen atoms of TC molecules and the unsaturated metals of the composite (Fe and Ce). Additionally, a π–π interaction was responsible for the adsorption of the aromatic structure of the TC molecule onto the (Ce_0.33_Fe) MIL-88A/10%g-C_3_N_4_. Furthermore, the π–π interaction could contribute to the TC adsorption onto the (Ce_0.33_Fe) MIL-88A/10%g-C_3_N_4_ surface since the N and O-containing catalyst can donate lone pairs of electrons to the π-orbitals of the TC molecules [4,50,51].

In the second stage, the degradation of TC occurs through an ^⦁^OH radical reaction, according to the results of the quenching experiment, as the presence of radical scavengers decreased the degradation efficiency. The XPS spectra of (Ce_0.33_Fe) MIL-88A/10%g-C_3_N_4_ before and after the TC degradation reaction were inspected to demonstrate the possible degradation mechanism. The Fe 2p spectrum of the used (Ce_0.33_Fe) MIL-88A/10%g-C_3_N_4_ catalyst exhibits a shift in the Fe 2p3/2 peaks from 710.79 and 713.16 eV to 710.82 and 713.36 eV, respectively. Also, the Fe 2p1/2 peaks demonstrate shifting from 724.04 and 727.08 eV to 724.18 and 716.95 eV, respectively (Figure 7C). Additionally, the Fe^3+^/Fe^2+^ ratio in the used catalyst incremented from 0.484 to 0.597, denoting the participation of Fe^2+^ in the activation of H_2_O_2_, as clarified in (Equation (11)) [12].

Likewise, the Ce^3+^ species involved in the activation of H_2_O_2_ (Equation (12)) since the associated peaks to Ce 3d5/2 after the Fenton-like degradation of TC slightly shifted, where the position of the Ce^3+^ peaks shifted from 882.38 and 886.02 eV to 882.6 and 886.12 eV, respectively, and also the belonging peaks to Ce^4+^ shifted from 889.64 and 895.53 eV to 890.08 and 895.63 eV, respectively, as clarified in (Figure 7D). Furthermore, the Ce 3d3/2 peaks of the Ce^3+^ species shifted slightly from 900.67 and 904.3 eV to 900.75 and 904.44 eV, and the position of the Ce^4+^ peak changed from 907.47 eV to 907.9 eV. Notably, the Ce^4+^/Ce^3+^ ratio in the (Ce_0.33_Fe) MIL-88A/10%g-C_3_N_4_ catalyst elevated from 0.207 to 0.265 after the TC degradation process.

**Figure 8:**
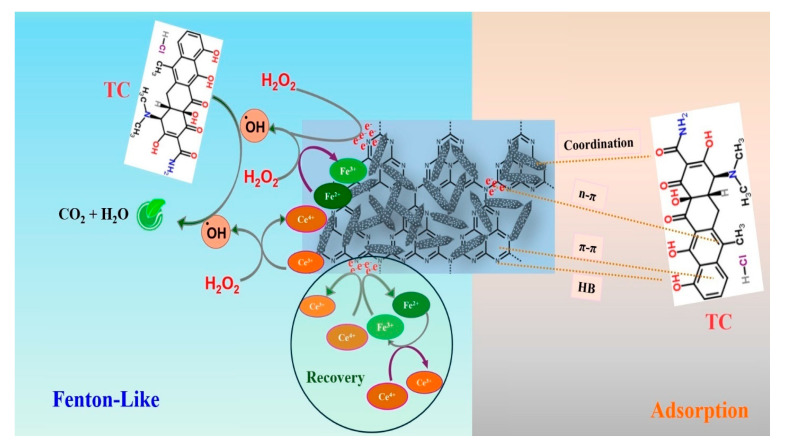
The schematic representation of the degradation of TC by (Ce_0.33_Fe) MIL-88A/10%g-C_3_N_4_ throughout the adsorption and Fenton-like processes.

Moreover, g-C_3_N_4_ possesses the ability to transfer charges, which assists in activating the H_2_O_2_ since it has the electron-rich heptazine ring structure of the pyridine nitrogen group, so it promotes the production of ^⦁^OH radicals. The shifting in the related peaks of nitrogen reflects the contribution of the g-C_3_N_4_ to the Fenton-like degradation process of TC (Figure 7E). Interestingly, these transferred electrons from g-C_3_N_4_ could recover the Fe^3+^ and Ce^4+^ ions [18,52]. More importantly, the regeneration of Ce^3+^ occurs by Fe^2+^, as elucidated in (Equation (13)), where the redox potential of Fe^3+^/Fe^2+^ (0.77 V) is considerably less than that of Ce^4+^/Ce^3+^ (1.44 V). The continuous formation of Fe^2+^ and Ce^3+^ ions by this continuous redox reaction can increase the quantities of ^⦁^OH produced by the breakdown of H_2_O_2_ and enhance the Fenton-like activity [53]. Ultimately, the generated ^⦁^OH by activating the H_2_O_2_ throughout the electron transfer from Fe^2+^, Ce^3+^, and g-C_3_N_4_ could degrade the TC molecules to less toxic molecules, as clarified in (Equation (14)). Figure 8 summarizes the possible degradation mechanism of the TC molecules via the contribution of adsorption and Fenton-like degradation processes.

(11) $${Fe}^{2+}+H_{2}O_{2}\to {Fe}^{3+}+{HO}^{•}+{HO}^{-}$$

(12) $${Ce}^{3+}+H_{2}O_{2}\to {Ce}^{4+}+{HO}^{•}+{HO}^{-}$$

(13) $${Fe}^{2+}+{Ce}^{4+}\to {Fe}^{3+}+{Ce}^{3+}$$

(14) $$TC+{HO}^{•}\to byproducts\to {CO}_{2}+H_{2}O$$

### 3.6. The Degradation Pathway of TC

**Figure 9:**
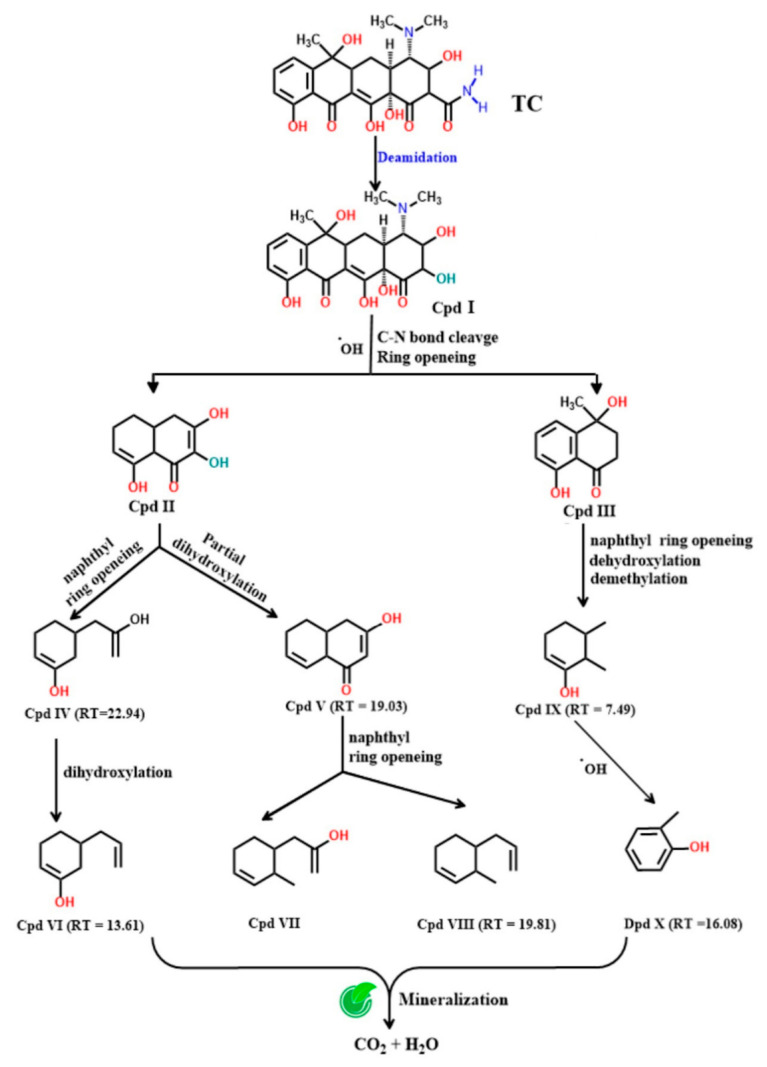
The yielded intermediates during the Fenton-like degradation of the TC molecules by (Ce_0.33_Fe) MIL-88A/10%g-C_3_N_4_.

The GC-MS spectrum (Figure S1) identified the intermediate compounds resulting from the degrading of the TC molecules. The previous studies demonstrated three main mechanisms of degradation: The loss of functional groups, ring-opening and central carbon cleavage, and hydroxylation reactions by adding or substituting ⦁OH. Based on the GC-MS findings, it was suggested the deamidation of TC to form Cpd I, as illustrated in Figure 9. The Cpd I underwent C–N bond cleavage and ring-opening to yield 2,3,8-trihydroxy-4,4a,5,6-tetrahydronaphthalen-1(8aH)-one (Cpd II) and 4,8-dihydroxy-4-methyl-3,4-dihydronaphthalen-1(2H)-one (Cpd III). Further degradation of Cpd II resulted in naphthyl ring-opening component 5-(2-hydroxyallyl) cyclohex-1-enol (Cpd IV) at R.T = 22.94 min, and the partial dihydroxylation compound 4,4a,5,6-tetrahydro-3-hydroxynaphthalen-1(8aH)-one (Cpd V) at R.T = 19.03 min. 5-allylcyclohex-1-enol (Cpd VI) at R.T = 13.61 min was generated by dihydroxylation of the Compound (Cpd IV). Further degradation of Cpd V resulted in the production of 3-(2-methylcyclohex-3-en-1-yl)prop-1-en-2-ol (Cpd VII) and 4-allyl-3-methylcyclohex-1-ene (Cpd VIII) at R.T = 19.81 min through a naphthyl ring opening. The Cpd III underwent naphthyl ring opening, dehydroxylation, and demethylation, producing 2,3-dimethylphenol (Cpd IX) at RT = 7.49 min that furtherly degraded to o-cresol (Cpd X) at RT = 16.08 min [54,55,56].

### 3.7. Recycling Test

**Figure 10:**
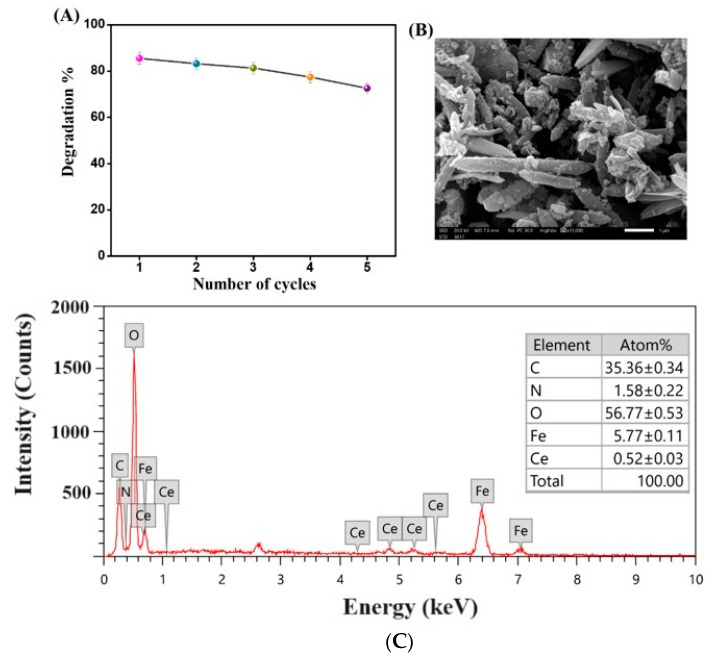
(**A**) Recycling test of the (Ce_0.33_Fe) MIL-88A/10%g-C_3_N_4_ catalyst during sequential five Fenton-like degradation cycles of the TC molecules, (**B**) SEM, and (**C**) SEM-EDX of the used (Ce_0.33_Fe) MIL-88A/10%g-C_3_N_4_ catalyst.

The recycling feature of the (Ce_0.33_Fe) MIL-88A/10%g-C_3_N_4_ catalyst was tested during sequential five Fenton-like degradation cycles of the TC molecules, as elucidated in (Figure 10A). Surprisingly, the degradation % of TC diminished from 85.52% to 72.56% after the 5th catalytic run. This promising result of the recycling test could be implied by the included continuous redox cycle in the (Ce_0.33_Fe) MIL-88A/10%g-C_3_N_4_ catalyst, endowing it with a self-recovery merit that elongates its lifetime.

### 3.8. Catalyst Stability

To prove the stability of (Ce_0.33_Fe) MIL-88A/10%g-C_3_N_4_ after the regeneration test, the catalyst was analyzed by SEM and SEM-EDX. The SEM Image of (Ce_0.33_Fe) MIL-88A/10%g-C_3_N_4_ (Figure 10B) showed no change in the catalyst morphology after the Fenton-like degradation reaction of the TC molecules, implying the promising stability of the as-synthesized Fenton-like catalyst. Furthermore, the SEM-EDX was utilized to define the metal leaching from (Ce_0.33_Fe) MIL-88A/10%g-C_3_N_4_ during the Fenton-like degradation of TC (Figure 10C). The findings demonstrate that the atomic % of the Fe and Ce species in the pure (Ce_0.33_Fe) MIL-88A/10%g-C_3_N_4_ are 7.96 and 0.85%, respectively. After the degradation, a slight metal leaching in Fe and Ce from (Ce_0.33_Fe) MIL-88A/10%g-C_3_N_4_ was recorded, reaching 5.77, and 0.52%, respectively. This observation is most likely due to the six nitrogen lone pairs-containing g-C_3_N_4_ that can be employed as electron donors to minimize the leaching of metal ions due to their distinct electrical characteristics and N-vacancy capabilities.

### 3.9. Comparison Study

A comparison study was conducted between the activity (Ce_0.33_Fe) MIL-88A/10%g-C_3_N_4_ catalyst and other previously reported Fenton-like catalysts towards degrading the TC molecules, as represented in Table 2. The comparison study clarified the high activity of the (Ce_0.33_Fe) MIL-88A/10%g-C_3_N_4_ catalyst during the Fenton-like degradation of TC using a low catalyst dose compared to the other heterogeneous catalysts.

## 4. Conclusions

This research developed a novel (Ce_0.33_Fe) MIL-88A/10%g-C_3_N_4_ material as a catalyst for TC degradation via a heterogeneous Fenton-like process. The SEM-EDX, FT-IR, XRD, XPS, BET, and SEM findings showed the morphological, compositional, and structural surface of (Ce_0.33_Fe) MIL-88A/10%g-C_3_N_4_. According to XRD measurements, sharp peaks indicate that the materials generated have crystalline characteristics. The SEM images represented supporting the hexagonal spindle (Ce_0.33_Fe) MIL-88A over the surface of the cracked sheet of g-C_3_N_4_. Under optimal conditions (pH = 7, catalyst dosage = 0.01 g, H_2_O_2_ = concentration 100 mg/L, temperature = 25 °C, and TC concentration = 50 mg/L), the (Ce_0.33_Fe) MIL-88A/10%g-C_3_N_4_/H_2_O_2_ system successfully degraded TC by 92.44% within 100 min. In the (Ce_0.33_Fe) MIL-88A/10%g-C_3_N_4_/H_2_O_2_ system, quenching experiments showed that ^•^OH is the principal ROS for TC degradation. The XPS analyses demonstrated a continuous redox cycle inside the active components of (Ce_0.33_Fe) MIL-88A/10%g-C_3_N_4_, producing plenty of electrons for the H_2_O_2_ activation and the generation of ^•^OH. The GC-MS pattern clarified the intermediate compounds from the TC degradation. The SEM-EDX suggested the stability of the (Ce_0.33_Fe) MIL-88A/10%g-C_3_N_4_ catalyst since the cerium and iron leaching concentrations after the Fenton-like degradation are inconsiderable. This finding explained the promising results of the recycling test that clarified a slight dwindle in the degradation % of TC after five catalytic cycles.

## Figures and Tables

**Table 1:** Derived parameter of kinetics study of the Fenton-like degradation of TC by (Ce_0.33_Fe) MIL-88A/10%g-C_3_N_4_ catalyst.

Kinetic Model	TC Concentrations (mg/L)			
	50	100	200	300
First order				
k1	0.0137	0.0052	0.0049	0.0036
R2	0.980	0.988	0.988	0.984
Second order				
k2	0.1511	0.0157	0.0128	0.0061
R2	0.828	0.973	0.979	0.962

**Table 2:** Comparison study between the catalytic efficiency of (Ce_0.33_Fe) MIL-88A/10%g-C_3_N_4_ catalyst and previous reported catalysts in relevant studies.

Catalysts	Parameters	Degradation %	Ref.
Fe-MPC	Time = 10 min, pH = 4.3, [H_2_O_2_] = 1 mM, [TC] = 40 mg/L, catalyst = 0.02 g.	83%	[57]
Fe_3_O_4_-Cs	Time = 120 min, pH = 3, [H_2_O_2_] = 10 mM, [TC] = 48.09 mg/L, catalyst = 0.5 g.	96%	[58]
MIL-101(Fe)@CS	Time = 140 min, pH = 5, [H_2_O_2_] = 0.09 mol/L, [TC] = 100 mg/L, catalyst = 2 g.	87.36%	[59]
S-mZVI/rGO	Time = 60 min, pH = 5, [H_2_O_2_] = 2 mM, [TC] = 20 mg/L, catalyst = 0.2 g.	97.2%	[60]
Fe/Cu-ZSM-5	Time = 10 min, pH = 7, [H_2_O_2_] = 1.0 g/L, [TC] = 30 mg/L, catalyst = 0.9 g.	99.02%	[61]
Fe_3_O_4_-S	Time = 110 min, pH = 7, [H_2_O_2_] = 50 mM, [TC] = 25 mg/L, catalyst = 0.5 g.	82.6%	[62]
CuCo-LDO/CN	Time = 40 min, pH = 6.9, [H_2_O_2_] = 15 mM, [TC] = 20 mg/L, catalyst = 0.08 g.	93.2%	[63]
IOT	Time = 360 min, pH = 3.6, [H_2_O_2_] = 1700 mg/L, [TC] = 200 mg/L, catalyst = 0.3 g.	95%	[64]
MnFe_2_O_4_@HL	Time = 120 min, pH = 3, [H_2_O_2_] = 1 mM, [TC] = 0.1 mM, catalyst = 0.3 g.	92.3%	[65]
Cu/CuFe_2_O_4_/DE	Time = 90 min, pH = 5, [H_2_O_2_] = 4.0 mM, [TC] = 50 mg/L, catalyst = 0.2 g.	95.5%	[66]
(Ce_0.33_Fe) MIL-88A/10%g-C_3_N_4_	Time = 100 min, pH = 7, [H_2_O_2_] = 100 mg/L, [TC] = 50 mg/L, catalyst = 0.01 g.	92.44%	This work

## Data Availability

Data will be available upon request.

## Supplementary Materials

The following supporting information can be downloaded at: https://www.mdpi.com/article/10.3390/nano14151282/s1, Figure S1: The GC-MS spectrum after the Fenton-like degradation of the TC molecules by (Ce_0.33_Fe) MIL-88A/10%g-C_3_N_4_.

## References

[1] El-Monaem E.M.A., Hosny M., Eltaweil A.S. (2024). Synergistic effect between adsorption and Fenton-like degradation on CoNiFe-LDH/ZIF-8 composite for efficient degradation of doxycycline. Chem. Eng. Sci..

[2] Omer A.M., El-Monaem E.M.A., El-Subruiti G.M., El-Latif M.M.A., Eltaweil A.S. (2021). Fabrication of easy separable and reusable MIL-125(Ti)/MIL-53(Fe) binary MOF/CNT/Alginate composite microbeads for tetracycline removal from water bodies. Sci. Rep..

[3] Abdelfatah A.M., Fawzy M., El-Khouly M.E., Eltaweil A.S. (2021). Efficient adsorptive removal of tetracycline from aqueous solution using phytosynthesized nano-zero valent iron. J. Saudi Chem. Soc..

[4] El-Monaem E.M.A., Omer A.M., Khalifa R.E., Eltaweil A.S. (2022). Floatable cellulose acetate beads embedded with flower-like zwitterionic binary MOF/PDA for efficient removal of tetracycline. J. Colloid Interface Sci..

[5] El-Monaem E.M.A., Eltaweil A.S., Elshishini H.M., Hosny M., Alsoaud M.M.A., Attia N.F., El-Subruiti G.M., Omer A.M. (2022). Sustainable adsorptive removal of antibiotic residues by chitosan composites: An insight into current developments and future recommendations. Arab. J. Chem..

[6] Fane A.G., Wang R., Hu M.X. (2015). Synthetic Membranes for Water Purification: Status and Future. Angew. Chem. Int. Ed..

[7] Shao S., Wu X. (2020). Microbial degradation of tetracycline in the aquatic environment: A review. Crit. Rev. Biotechnol..

[8] Tang S., Zhao M., Yuan D., Li X., Wang Z., Zhang X., Jiao T., Ke J. (2021). Fe_3_O_4_ nanoparticles three-dimensional electro-peroxydisulfate for improving tetracycline degradation. Chemosphere.

[9] Wang W., Li Z., Wu K., Dai G., Chen Q., Zhou L., Zheng J., Ma L., Li G., Wang W. (2023). Novel Ag-bridged dual Z-scheme g-C3N4/BiOI/AgI plasmonic heterojunction: Exceptional photocatalytic activity towards tetracycline and the mechanism insight. J. Environ. Sci..

[10] Teng Y., Li W., Wang J., Jia S., Zhang H., Yang T., Li X., Li L., Wang C. (2023). A green hydrothermal synthesis of polyacrylonitrile@carbon/MIL-101(Fe) composite nanofiber membrane for efficient selective removal of tetracycline. Sep. Purif. Technol..

[11] Abbasifar A., Shahrabadi F., ValizadehKaji B. (2020). Effects of green synthesized zinc and copper nano-fertilizers on the morphological and biochemical attributes of basil plant. J. Plant Nutr..

[12] Liao X., Wang F., Wang F., Cai Y., Yao Y., Teng B.-T., Hao Q., Shuxiang L. (2019). Synthesis of (100) surface oriented MIL-88A-Fe with rod-like structure and its enhanced fenton-like performance for phenol removal. Appl. Catal. B Environ..

[13] Ren G., Zhao K., Zhao L. (2020). A Fenton-like method using ZnO doped MIL-88A for degradation of methylene blue dyes. RSC Adv..

[14] Zhu Y., Zhu R., Xi Y., Zhu J., Zhu G., He H. (2019). Strategies for enhancing the heterogeneous Fenton catalytic reactivity: A review. Appl. Catal. B Environ..

[15] Liu C., Wang P., Huang P., Yang Z., Zhou G. (2023). Photo-induced heterogeneous regeneration of Fe(II) in Fenton reaction for efficient polycyclic antibiotics removal and in-depth charge transfer mechanism. J. Colloid Interface Sci..

[16] Liu L., Yu R., Zhao S., Cao X., Zhang X., Bai S. (2023). Heterogeneous Fenton system driven by iron-loaded sludge biochar for sulfamethoxazole-containing wastewater treatment. J. Environ. Manag..

[17] Tang J., Wang J. (2018). Fenton-like degradation of sulfamethoxazole using Fe-based magnetic nanoparticles embedded into mesoporous carbon hybrid as an efficient catalyst. Chem. Eng. J..

[18] Li T., Ge L., Peng X., Wang W., Zhang W. (2021). Enhanced degradation of sulfamethoxazole by a novel Fenton-like system with significantly reduced consumption of H_2_O_2_ activated by g-C3N4/MgO composite. Water Res..

[19] Wang J., Tang J. (2021). Fe-based Fenton-like catalysts for water treatment: Preparation, characterization and modification. Chemosphere.

[20] Omer A.M., El-Monaem E.M.A., El-Latif M.M.A., El-Subruiti G.M., Eltaweil A.S. (2021). Facile fabrication of novel magnetic ZIF-67 MOF@aminated chitosan composite beads for the adsorptive removal of Cr(VI) from aqueous solutions. Carbohydr. Polym..

[21] Zhao B., Gong J., Song B., Sang F., Zhou C., Zhang C., Cao W., Niu Q., Chen Z. (2022). Effects of activated carbon, biochar, and carbon nanotubes on the heterogeneous Fenton oxidation catalyzed by pyrite for ciprofloxacin degradation. Chemosphere.

[22] El-Monaem E.M.A., Eltaweil A.S., El-Subruiti G.M., Mohy-Eldin M.S., Omer A.M. (2023). Adsorption of nitrophenol onto a novel Fe3O4-κ-carrageenan/MIL-125(Ti) composite: Process optimization, isotherms, kinetics, and mechanism. Environ. Sci. Pollut. Res..

[23] Xu Y., Rashwan A.K., Osman A.I., El-Monaem E.M.A., Elgarahy A.M., Eltaweil A.S., Omar M., Li Y., Mehanni A.-H.E., Chen W. (2023). Synthesis and potential applications of cyclodextrin-based metal–organic frameworks: A review. Environ. Chem. Lett..

[24] Ai L., Zhang C., Li L., Jiang J. (2014). Iron terephthalate metal–organic framework: Revealing the effective activation of hydrogen peroxide for the degradation of organic dye under visible light irradiation. Appl. Catal. B Environ..

[25] Lv H., Zhao H., Cao T., Qian L., Wang Y., Zhao G. (2015). Efficient degradation of high concentration azo-dye wastewater by heterogeneous Fenton process with iron-based metal-organic framework. J. Mol. Catal. A Chem..

[26] Akgün D., Dükkancı M. (2023). g-C_3_N_4_ supported Ag/AgCl@MIL-88A MOF based triple composites for highly efficient diuron photodegradation under visible LED light irradiation. J. Water Process Eng..

[27] Ahmad M., Quan X., Chen S., Yu H. (2020). Tuning Lewis acidity of MIL-88B-Fe with mix-valence coordinatively unsaturated iron centers on ultrathin Ti_3_C_2_ nanosheets for efficient photo-Fenton reaction. Appl. Catal. B Environ..

[28] Xu H., Shan C., Wu X., Sun M., Huang B., Tang Y., Yan C.-H. (2020). Fabrication of layered double hydroxide microcapsules mediated by cerium doping in metal–organic frameworks for boosting water splitting. Energy Environ. Sci..

[29] Yu D., Wang L., Yang T., Yang G., Wang D., Ni H., Wu M. (2021). Tuning Lewis acidity of iron-based metal-organic frameworks for enhanced catalytic ozonation. Chem. Eng. J..

[30] Ge L., Peng Z., Wang W., Tan F., Wang X., Su B., Qiao X., Wong P.K. (2018). g-C3N4/MgO nanosheets: Light-independent, metal-poisoning-free catalysts for the activation of hydrogen peroxide to degrade organics. J. Mater. Chem. A.

[31] Su S., Xing Z., Zhang S., Du M., Wang Y., Li Z., Chen P., Zhu Q., Zhou W. (2021). Ultrathin mesoporous g-C3N4/NH2-MIL-101(Fe) octahedron heterojunctions as efficient photo-Fenton-like system for enhanced photo-thermal effect and promoted visible-light-driven photocatalytic performance. Appl. Surf. Sci..

[32] Wang Y., Fang Q., Xie Z., Hu C., Lyu L. (2022). Enhanced Fenton-like process via interfacial electron donating of pollutants over in situ Cobalt-doped graphitic carbon nitride. J. Colloid Interface Sci..

[33] Wang Z., Wang J., Iqbal W., Shi M., Yang L., Chang N., Qin C. (2023). Morphology-effects of four different dimensional graphitic carbon nitrides on photocatalytic performance of dye degradation, water oxidation and splitting. J. Phys. Chem. Solids.

[34] Gao M., Li B., Liu J., Hu Y., Cheng H. (2023). Adsorption behavior and mechanism of p-arsanilic acid on a Fe-based metal–organic framework. J. Colloid Interface Sci..

[35] Li N., Du H., Tan M., Yang L., Xue B., Zheng S., Wang Q. (2023). Construction of Z-scheme CuBi2O4/MIL-88A(Fe) heterojunctions with enhanced LED light driven photocatalytic Cr(VI) reduction and antibacterial performance. Appl. Surf. Sci..

[36] Yang B., Dai J., Zhao Y., Wang Z., Wu J., Ji C., Zhang Y., Pu X. (2023). Synergy effect between tetracycline and Cr(VI) on combined pollution systems driving biochar-templated Fe_3_O_4_@SiO_2_/TiO_2_/g-C_3_N_4_ composites for enhanced removal of pollutants. Biochar.

[37] Ke Y., You Q., Ai J., Yang X., Shang Q., Liu Y., Wang D., Liao G. (2023). Improved performance of visible-light photocatalytic H2-production and Cr(VI) reduction by waste pigeon guano doped g-C_3_N_4_ nanosheets. J. Mater. Sci. Technol..

[38] Li M., Li C., Chunrui Z., Li T., Jiang J., Han Z., Zhang C., Sun H., Dong S. (2023). Citric acid-modified MIL-88A(Fe) for enhanced photo-Fenton oxidation in water decontamination. Sep. Purif. Technol..

[39] Yao Y., Zheng H., Tao Z., Wang Y., Ma Z., Qiu Y., Wang S. (2023). Highly efficient oxidation of aqueous contaminants by mesoporous graphitic carbon nitride supported copper phosphide with dual active sites. Appl. Surf. Sci..

[40] Tang C., Ma S.-Q., Yi X.-H., Wang C.-C., Wang P. (2023). MIL-88A(Fe)/TCNQ composites for boosted photo-Fenton sulfamethoxazole degradation under LED visible light. Mater. Res. Bull..

[41] Chen J., Qin C., Mou Y., Cao Y., Chen H., Yuan X., Wang H. (2023). Linker regulation of iron-based MOFs for highly effective Fenton-like degradation of refractory organic contaminants. Chem. Eng. J..

[42] Gogoi A., Navgire M., Sarma K.C., Gogoi P. (2019). Highly efficient heterogeneous Fenton activities of magnetic β-cyclodextrin (Fe) framework for Eriochrome black T degradation. Mater. Chem. Phys..

[43] Seo J., Gowda A., Babu S. (2018). Almost Complete Removal of Ceria Particles Down to 10 nm Size from Silicon Dioxide Surfaces. ECS J. Solid State Sci. Technol..

[44] Vazirov R.A., Sokovnin S.Y., Ilves V.G., Bazhukova I.N., Pizurova N., Kuznetsov M.V. (2018). Physicochemical characterization and antioxidant properties of cerium oxide nanoparticles. J. Phys. Conf. Ser..

[45] El-Monaem E.M.A., Ayoup M.S., Omer A.M., Hammad E.N., Eltaweil A.S. (2023). Sandwich-like construction of a new aminated chitosan Schiff base for efficient removal of Congo red. Appl. Water Sci..

[46] Li L., Yang S., Wang Y., Hui S., Xiao T., Kong J., Zhao X. (2023). Nitrogen-doped carbon nanosheets for efficient degradation of bisphenol A by H_2_O_2_ activation at neutral pH values. Sep. Purif. Technol..

[47] Du Z., Zhou C., Chen D., Li W., Wang H., Wu H., Zhang Z., Yang H. (2023). Coupling of gaseous toluene adsorption and heterogeneous oxidative degradation by iron-based porous polymeric resin LXQ-10. Fuel.

[48] Chen Y., Zhang M., Chen T., Zhang G., Xu H., Sun H., Zhang L. (2023). Facile fabrication of rGO/PPy/nZVI catalytic microreactor for ultrafast removal of p-nitrophenol from water. Appl. Catal. B Environ..

[49] Eltaweil A.S., Bakr S.S., El-Monaem E.M.A., El-Subruiti G.M. (2023). Magnetic hierarchical flower-like Fe_3_O_4_@ZIF-67/CuNiMn-LDH catalyst with enhanced redox cycle for Fenton-like degradation of Congo red: Optimization and mechanism. Environ. Sci. Pollut. Res..

[50] Ashrafi M., Farhadi S. (2023). Polyoxometalate supported on a magnetic Fe_3_O_4_/MIL-88A rod-like nanocomposite as an adsorbent for the removal of ciprofloxacin, tetracycline and cationic organic dyes from aqueous solutions. RSC Adv..

[51] Zhang Z., Chen Y., Wang Z., Hu C., Ma D., Chen W., Ao T. (2021). Effective and structure-controlled adsorption of tetracycline hydrochloride from aqueous solution by using Fe-based metal-organic frameworks. Appl. Surf. Sci..

[52] Su L., Wang P., Li M., Zhao Z., Li Y., Zhan S. (2023). Synergistic enhancement of photocatalytic molecular oxygen activation by nitrogen defect and interfacial photoelectron transfer over Z-scheme α-Fe_2_O_3_/g-C3N4 heterojunction. Appl. Catal. B Environ..

[53] Ghorai K., Panda A., Bhattacharjee M., Mandal D., Hossain A., Bera P., Seikh M.M., Gayen A. (2021). Facile synthesis of CuCr_2_O_4_/CeO_2_ nanocomposite: A new Fenton like catalyst with domestic LED light assisted improved photocatalytic activity for the degradation of RhB, MB and MO dyes. Appl. Surf. Sci..

[54] Balu K., Chicardi E., Sepúlveda R., Durai M., Ishaque F., Chauhan D., Ahn Y.-H. (2023). BiOX (X = I or Cl?) modified Na-K2Ti_6_O_13_ nanostructured materials for efficient degradation of Tetracycline, Acid Black 1 dye and microbial disinfection in wastewater under Blue LED. Sep. Purif. Technol..

[55] Beena J., Joji L.R. (2015). Analysis of the Essential Oil from the Leaves of *Wrightia tinctoria* R. Br. from South India. Int. J. Ayurveda Pharma Res..

[56] Limam I., Guenne A., Driss M., Mazeas L. (2010). Simultaneous determination of phenol, methylphenols, chlorophenols and bisphenol-A by headspace solid-phase microextraction-gas chromatography-mass spectrometry in water samples and industrial effluents. Int. J. Environ. Anal. Chem..

[57] Wang C., Sun R., Huang R., Wang H. (2021). Superior fenton-like degradation of tetracycline by iron loaded graphitic carbon derived from microplastics: Synthesis, catalytic performance, and mechanism. Sep. Purif. Technol..

[58] Li X., Cui K., Guo Z., Yang T., Cao Y., Xiang Y., Chen H., Xi M. (2020). Heterogeneous Fenton-like degradation of tetracyclines using porous magnetic chitosan microspheres as an efficient catalyst compared with two preparation methods. Chem. Eng. J..

[59] Zhang A., Shan F., Zhang Z., Wang J., Zhang T., Liu M. (2024). Efficient decontamination of tetracycline via fenton-like process mediated by chitosan-based Fe-MOFs under wide pH range. Sep. Purif. Technol..

[60] Zhang X., Sun W., Zhang X., Wang Y., Li T., Wang H. (2024). Mechanochemical synthesis of sulfidated microscale zerovalent iron/graphene composite for enhanced degradation of tetracycline. Sep. Purif. Technol..

[61] Zhao Y., Gu S., Li L., Wang M. (2024). From waste to catalyst: Growth mechanisms of ZSM-5 zeolite from coal fly ash & rice husk ash and its performance as catalyst for tetracycline degradation in fenton-like oxidation. Environ. Pollut..

[62] Nie M., Li Y., He J., Xie C., Wu Z., Sun B., Zhang K., Kong L., Liu J. (2020). Degradation of tetracycline in water using Fe3O4 nanospheres as Fenton-like catalysts: Kinetics, mechanisms and pathways. New J. Chem..

[63] Gu Y.-Y., Wu Z., Shen Y., Lu C., Lu L., Bian Z., Zhang X., Zhao C., Fu R., Li H. (2023). Efficient Fenton-like degradation of tetracycline by stalactite-like CuCo-LDO/CN catalysts: The overlooked contribution of dissolved oxygen. Chemosphere.

[64] Tian G., Nie K., Han G., Hua X., Zhang K., Dang S., Yao B., Meng J. (2023). Iron ore tailings as efficient Fenton-like catalysts for degradation of tetracycline. Mater. Today Commun..

[65] Zhao W., Yang B. (2024). Fabrication of magnetic MnFe_2_O_4_@HL composites with an in situ Fenton-like reaction for enhancing tetracycline degradation. J. Colloid Interface Sci..

[66] Deng S., Guo Z., Chen Y.-H., Cui K.-P., Ding Z.-G., Wang B., Weerasooriya R., Chen X. (2023). Fabrication of Cu0-composited CuFe_2_O_4_ magnetic nanoparticles on diatomite support for efficient degradation of tetracycline hydrochloride by a Fenton-like system. J. Environ. Chem. Eng..

