# Supplementary material for: Enhanced Fenton Degradation of Tetracycline over Cerium-Doped MIL88-A/g-C3N4: Catalytic Performance and Mechanism
Source: Nanomaterials (Basel). 2024 Jul 30;14(15):1282. doi: 10.3390/nano14151282 (PMC11313986; doi:10.3390/nano14151282)

Enhanced Fenton degradation of tetracycline over Cerium-doped MIL88-A/g-C_3N_4: catalytic performance, and mechanism

Abdelazeem S. Eltaweil [1,2], Amira M. Galal [2] and Eman M. Abd El-Monaem [2], Nouf Al Harby [3,*], Mervette El Batouti [2]

Figure S1. The GC-MS spectrum after the Fenton-like degradation of the TC molecules by

($Ce_{0.33}Fe$) MIL-88A/10%g-C_3N_4.

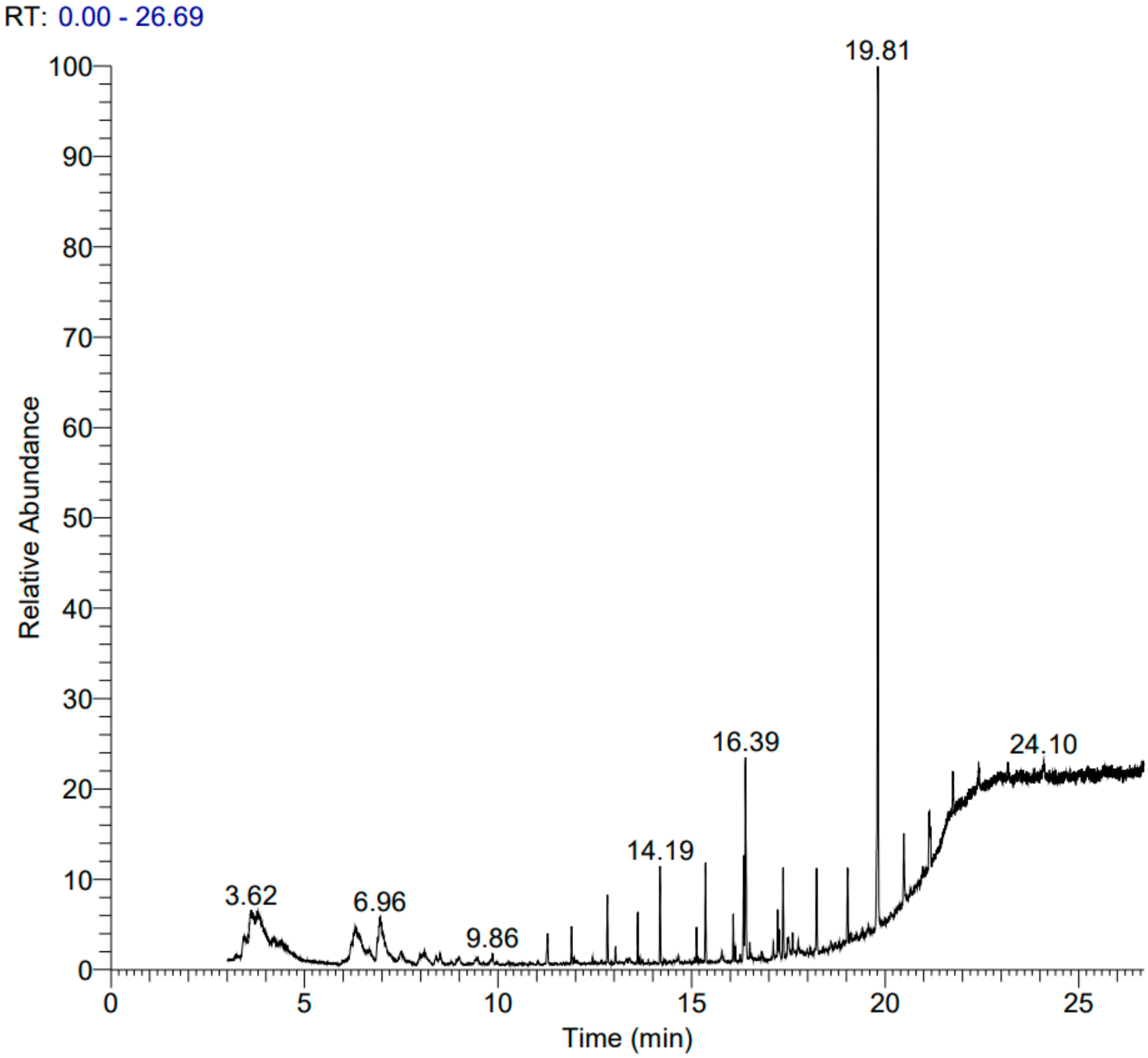

Supplement: Supplementary file 1 [file nanomaterials-14-01282-s001.zip › nanomaterials-3098153-supplementary.pdf]
